# Development of a competitive chemiluminescence immunoassay using a monoclonal antibody recognizing 3B of foot-and-mouth disease virus for the rapid detection of antibodies induced by FMDV infection

**DOI:** 10.1186/s12985-021-01663-4

**Published:** 2021-09-26

**Authors:** Wei Liu, Guanglei Zhang, Sicheng Yang, Junhui Li, Zhan Gao, Sudan Ge, Huihui Yang, Junjun Shao, Huiyun Chang

**Affiliations:** State Key Laboratory of Veterinary Etiological Biology, National Foot-and-Mouth Diseases Reference Laboratory, Lanzhou Veterinary Research Institute, Chinese Academy of Agricultural Sciences, Lanzhou, Gansu China

**Keywords:** Chemiluminescence immunoassay, Diagnosis, Foot-and-mouth disease virus, Monoclonal antibody, Non-structural protein

## Abstract

**Background:**

Foot-and-mouth disease (FMD) is a devastating animal disease. Anti-non-structural protein (NSP) antibody detection is very important for confirming suspected cases, evaluating the prevalence of infection, certifying animals for trade and controlling the disease.

**Methods:**

In this study, a competitive chemiluminescence immunoassay (3B-cCLIA) was developed for the rapid detection of antibodies against NSPs in different species of livestock animals using the monoclonal antibody (mAb) 9E2 as a competitive antibody that recognizes NSP 3B.

**Results:**

The cut-off value (50%), diagnostic sensitivity (Dsn) (97.20%, 95.71%, and 96.15%) and diagnostic specificity (Dsp) (99.51%, 99.43%, and 98.36) of the assay were estimated by testing a panel of known-background sera from swine, cattle and sheep, respectively. The accuracy rate of the 3B-cCLIA was further validated and subsequently compared with that of two commercial diagnostic kits. The early diagnostic results showed that antibodies recognizing NSPs developed later (approximately 1–2 days) than antibodies recognizing structural proteins. Furthermore, anti-NSP antibody presence in animals vaccinated multiple times (false positives), especially cattle and sheep, was confirmed, and the false-positive rate increased with the number of vaccinations.

**Conclusions:**

These results indicate that the 3B-cCLIA is suitable for the rapid detection of antibodies against FMDV NSP 3B in a wide range of species.

**Supplementary Information:**

The online version contains supplementary material available at 10.1186/s12985-021-01663-4.

## Background

Foot-and-mouth disease (FMD) is a highly contagious and economically damaging viral disease that affects cloven-hoofed animals. FMD virus (FMDV) has positive-sense, single-stranded RNA genome that encodes four structural proteins (SPs: VP4, VP2, VP3 and VP1) and ten non-structural proteins (NSPs: L, 2A, 2B, 2C, 3A, 3B, 3C, 3D, 3AB and 3ABC) [1–3]. FMDV exists in the form of seven serotypes (A, O, C, Asia 1, SAT 1, SAT 2, and SAT 3); serotypes O and A are currently prevalent in China [4, 5].

To date, slaughtering infected and contacted animals and prohibiting the importation of animals and animal products from FMD-endemic countries are practised to prevent the disease in FMD-free nations. Considering the economic costs, vaccination policies have been adopted for the control and eradication of the disease in endemic countries [6, 7]. However, vaccination with inactivated vaccines creates other issues, such as the differentiation between FMDV-infected and vaccinated animals (DIVA) and the creation of carrier animals that shed the virus [6, 7]. Therefore, the detection of antibodies against NSPs has become a widely preferred and applied diagnostic method for DIVA that is helpful in identifying subclinical infections, evaluating the prevalence of infection and controlling the disease [8] because a series of purifying techniques remove the majority of NSPs from the inactivated vaccine during production [3, 9].

There are two main diagnostic methods for the detection of antibodies against NSPs. One method is an indirect ELISA based on recombinant NSPs, peptides or epitopes [1, 8, 10–15]. Species-specific conjugates are needed for indirect ELISAs, which makes simultaneous examination of sera from different species difficult [16]. Another method is a blocking or competitive ELISA using polyclonal antibodies or monoclonal antibodies (mAbs) [6, 9, 16–20]. In contrast to indirect ELISAs, blocking or competitive ELISAs can be used to detect antibodies in each species that is susceptible to FMD. Most commercial ELISAs are blocking ELISAs, such as the 3ABC monoclonal antibody blocking ELISA (3ABC-bELISA, LVRI, China) and PrioCHECK FMDV NSP ELISA.

In a previous study, the mAb 9E2 recognizing NSP 3B that can be used to develop a diagnostic method was preliminarily verified. However, the mAb 2G5 recognizing NSP 3A alone is not suitable for the establishment of a diagnostic method because of mutations and deletions in the epitope identified by the mAb 2G5 [3]. In this study, we used the mAb 9E2 as the detection antibody to develop a competitive chemiluminescence immunoassay (termed 3B-cCLIA) for the rapid detection of anti-NSP antibodies. To decrease the false positive rate in cattle and sheep that had been vaccinated multiple times, we attempted to use two mAbs recognizing NSP 3A and 3B to develop a 3A + 3B-cCLIA.

## Materials and methods

### Serum samples

Serum samples from naïve animals: Serum samples from clinically healthy and unvaccinated animals, including 310, 175, and 61 samples from swine, cattle, and sheep, respectively, were collected and tested using a liquid-phase blocking ELISA for FMDV O (O-LPBE) and A-LPBE (with negative results; titer, < 1:4). The diagnostic specificity (Dsp) and cut-off value were calculated using these serum samples (Additional file 2: Table S1).

Serum samples from infected animals: The collection of 107 serum samples from swine infected with FMDV A/GDMM/2013 or O/Mya98 at 7–25 days post infection (dpi) was carried out in the Animal Biological Safety Level 3 (ABSL-3) Laboratory at Lanzhou Veterinary Research Institute (Lanzhou, China); 70 serum samples were collected from cattle infected with FMDV (A/GDMM/2013 or O/Mya98) at 8–20 dpi, and 52 serum samples were collected from sheep with clinical symptoms in the field, which tested as NSP positive using two commercial diagnostic kits (3ABC-bELISA and PrioCHECK FMDV NSP ELISA). The diagnostic sensitivity (Dsn) and cut-off value were calculated using these samples. In addition, a total of 32 serum samples collected from four unvaccinated control swine experimentally challenged with FMDV O/Mya98 at 0 and 2–8 dpi were used to compare the early diagnostic performance of the 3B-cCLIA and two commercial diagnostic kits and detect seroconversion to FMDV SPs and NSPs (Additional file 2: Table S1).

Serum samples from vaccinated animals: One hundred serum samples were collected at 21 days post vaccination (dpv) from swine vaccinated with an FMDV O univalent multiple-epitope recombinant vaccine developed by our research group. The Dsp and cut-off value were calculated using these samples. In addition, 120 serum samples were collected from sows vaccinated 3–15 times with commercial O/A divalent inactivated vaccines. Similarly, 129 serum samples were collected from dairy cows vaccinated with commercial O/A divalent inactivated vaccines every four months for a total of 2, 5 or 10 vaccinations. Seventy-seven serum samples were collected from 15 sheep vaccinated 1–3 times with the laboratory-made FMDV A/AF72, O/Mya98/BY/2010, or Asia 1/JS05 univalent inactivated vaccine. These samples were used to evaluate the diagnostic performances of the 3B-cCLIA and two commercial diagnostic kits and verify the false-positive phenomenon in animals vaccinated multiple times (Additional file 2: Table S1).

Serum samples collected in the field: One hundred seventy-three serum samples were collected from field swine suspected of FMDV infection during 2010–2018. These sera were used to compare the coincidence rates between the 3B-cCLIA and two commercial diagnostic kits (Additional file 2: Table S1).

Serum samples from other virus-infected swine: In this study, one serum sample from a classical swine fever virus (CSFV)-infected swine, one serum sample from a Senecavirus A (SVA)-infected swine, one serum sample from a porcine parvovirus (PPV)-infected swine, one serum sample from a porcine reproductive and respiratory syndrome virus (PRRSV)-infected swine, and two serum samples from porcine circovirus type 2 (PCV2)-infected swine were investigated (Additional file 2: Table S1).

Control sera: A serum sample derived from swine infected with FMDV O/Mya98 at 25 dpi served as standard positive serum (P51). The percentage inhibition (PI) rates for the 3ABC-bELISA and PrioCHECK FMDV NSP ELISA were 92% and 93%, respectively. A standard negative serum sample (P734) was taken from a clinically healthy swine that had not been immunized against FMD. The serum was tested with the O-LPBE and A-LPBE (with negative results; titer, < 1:4), 3ABC-bELISA (with a negative result; PI = 1%), and PrioCHECK FMDV NSP ELISA (with a negative result; PI = −7%).

### Antigen and antibodies

The 3ABC-coding region of FMDV A/GDMM/2013 mutated at positions 46 aa and 163 aa was cloned into the pProEXHTB plasmid. The expression and purification of the recombinant 3ABC protein has been described elsewhere [13].

MAbs against the 3ABC protein, designated 2G5 and 9E2, were obtained in our laboratory, and their minimally identified epitopes were “^92^EYIEKA^97^”, which is located in 3A, and “^23^EGPYAGPLE^31^”, which is located in 3B [3]. The mAbs 2G5 and 9E2 were largely produced by injecting hybridomas into the peritoneal cavities of BALB/c mice and purified by affinity protein G column chromatography. Then, the mAbs 2G5 and 9E2 were conjugated with horseradish peroxidase (HRP). Polyclonal antibodies were obtained by inoculating rabbits with the purified 3ABC protein.

### Development of a competitive CLIA using a mAb against NSP 3B

Checkerboard titration was performed to optimize the conditions of the 3B-cCLIA. Coating with the mAb 2G5 on 96-well white plates (Costar, catalogue number: 43923) was carried out at 1, 0.5, 0.25, and 0.125 μg/mL concentrations in a 100-μL volume followed by overnight incubation at 4 °C. After washing, purified 3ABC protein was diluted to 0.5, 0.25, and 0.125 μg/mL in PBS containing 0.05% Tween-20 (PBST) and added to each well, and the plate was incubated for 1 h at 37 °C. After three PBST washes, each well received 200 μL of blocking buffer, followed by incubation at 37 °C for 2 h. Then, serial dilutions (1:2.5–1:20) of standard positive serum (P51) and standard negative serum (P734) were carried out with dilution buffer (10% equine serum, 1% casein in PBST), and 50 μL of serum was transferred to each well. Simultaneously, 50 μL of HRP-conjugated 9E2 (9E2-HRP) was added to each well at concentrations of 0.08, 0.04, 0.02, 0.01, 0.005, and 0.0025 μg/mL, followed by incubation of the plate at room temperature. After washing five times, a chemiluminescence (CL) substrate (KEY-BIO, Beijing, China) including 50 μL of solution A (luminol and luminous enhancer) and 50 μL of solution B (peroxide solution) was added. The CL signals were measured with a Varioskan LUX (Thermo Scientific, USA) after 5 min. Determination of the optimum mAb 2G5 concentration, 3ABC protein concentration, serum dilution, 9E2-HRP concentration, and incubation time was carried out based on the ratios of the CL values of standard negative serum to those of standard positive serum (N/P).

The 3B-cCLIA was carried out under optimal conditions. The following formula was used to calculate the PI:

$${\text{PI}}\% = \left( {1 - {\text{CLs/CLn}}} \right) \times 100\% ,$$

where the mean CL value of the standard negative serum control is represented by CLn, while the CL values of the test samples are represented by CLs.

A mean CLn ≥ 6,000,000 and mean PI of the standard positive control > 80% indicated assay validity.

Similarly, the 3A + 3B-cCLIA was developed; detailed information is presented in Additional file 1.

### Cut-off value, Dsn, and Dsp

The cut-off value of the 3B-cCLIA was determined by testing 875 serum samples from different origins. NSP-negative sera from swine (n = 410), cattle (n = 175), and sheep (n = 61) were used to estimate the Dsp for each species, and NSP-positive sera from swine (n = 107), cattle (n = 70), and sheep (n = 52) were used to estimate the Dsn for each species using MedCalc software.

### Comparison of accuracy rates and diagnostic performances

The accuracy rates of two commercial diagnostic kits were also evaluated using the abovementioned 875 NSP-negative and NSP-positive serum samples. The diagnostic performances of the 3B-cCLIA, 3A + 3B-cCLIA and two commercial diagnostic kits were compared by testing 430 serum samples, including 120, 60, and 77 samples from vaccinated swine, cattle, and sheep, respectively, and 173 samples from the field. In addition, serum samples from four swine challenged with FMDV O/Mya98 collected at different times were utilized to evaluate and compare the early diagnostic performances of the four assays.

### Estimation of the repeatability and stability of the 3B-cCLIA

To calculate the intra- and interbatch repeatability performances, three replicates of seven serum samples with varying PI values were evaluated on various days using plates coated in the same and different batches. To determine the shelf life of the coated plates, they were vacuum packed and stored at 37 °C for 15 days or at 4 °C for 1 year after blocking.

## Results

### Optimization of the 3B-cCLIA

The reaction conditions were optimized using checkerboard titration to obtain high N/P ratios. In the 3B-cCLIA, the coating concentrations of the mAb 2G5 and 3ABC protein were fixed at 0.25 μg/mL, the serum dilution was 1:2.5, the concentration of 9E2-HRP was 0.005–0.02 μg/mL (0.01 μg/mL), and the reaction time was 10 min.

### Determination of the cut-off value, Dsn, and Dsp for the 3B-cCLIA

**Fig. 1:**
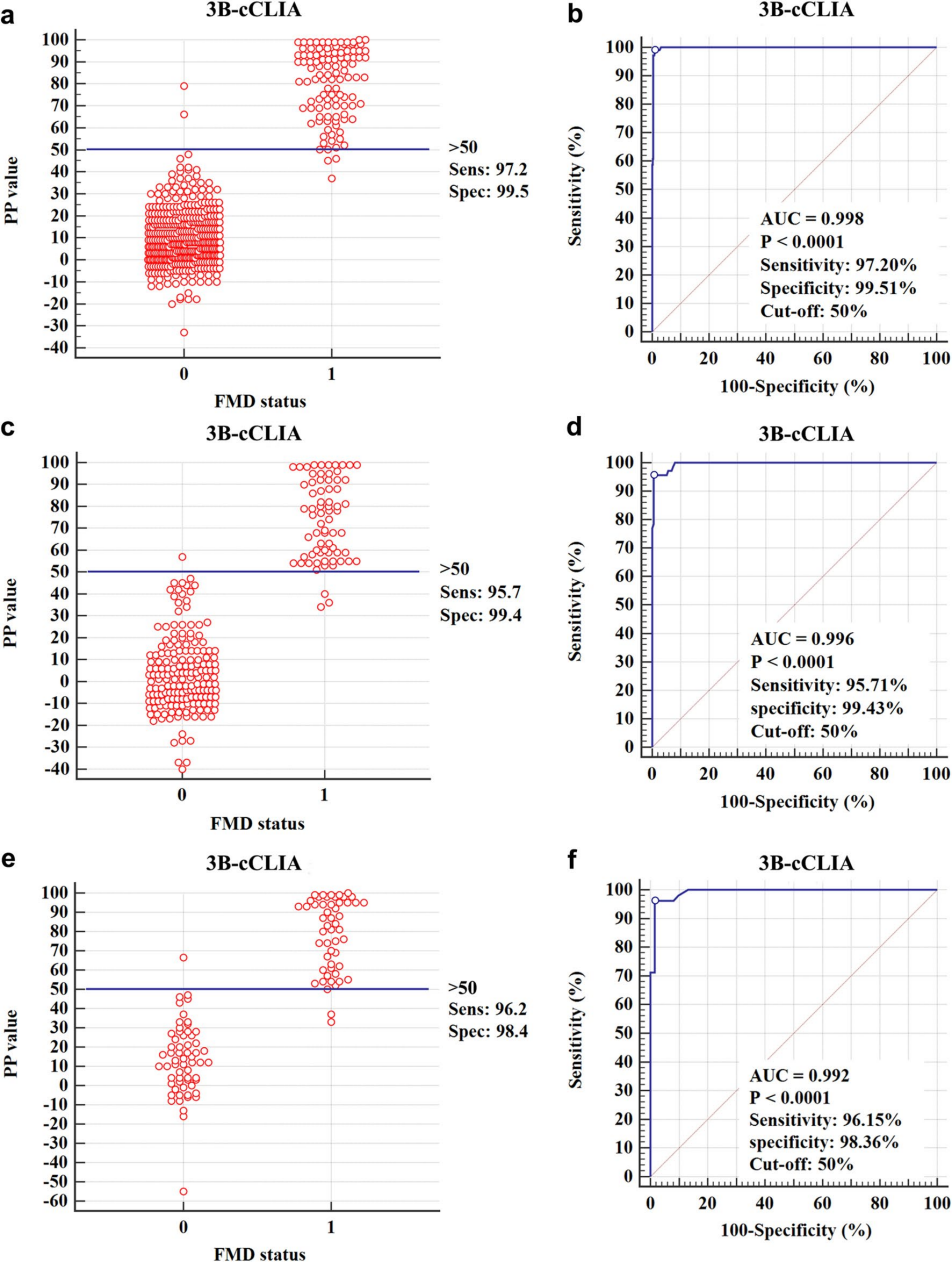
Receiver operating characteristic (ROC) analysis for the determination of the cut-off value of the 3B-cCLIA. **a**, **c**, **e** Interactive dot diagram of the 3B-cCLIA in testing sera from swine, cattle, and sheep. 0, negative serum samples (n = 410, 175, and 61); 1, positive serum samples (n = 107, 70, and 52). **b**, **d**, **f** Each point on the ROC curve represents a sensitivity–specificity pair in testing the sera from swine, cattle, and sheep

The cut-off values for the 3B-cCLIA were determined using known-background sera from swine (NSP-negative sera, n = 410; NSP-positive sera, n = 107), cattle (NSP-negative sera, n = 175; NSP-positive sera, n = 70) and sheep (NSP-negative sera, n = 61; NSP-positive sera, n = 52) by analysing the interactive dot diagram and receiver operating characteristic (ROC) curve (Fig. 1 and Table 1). According to the analysis of the ROC curve, when the cut-off for the 3B-cCLIA was 50%, the Dsn was 97.20% (104/107), 95.71% (67/70), and 96.15% (50/52), and the Dsp was 99.51% (408/410), 99.43% (174/175), and 98.36% (60/61) in swine, cattle, and sheep, respectively. Furthermore, cross-reaction evaluation of the 3B-cCLIA with sera from different virus-infected swine (CSFV, SVA, PPV, PRRSV, and PCV2) revealed no cross-reactivity (PI < 20%).

**Table 1:** Comparison of 3B-cCLIA with 3A + 3B-cCLIA, 3ABC-bELISA and PrioCHECK FMDV NSP ELISA for the detection of NSP antibodies in sera from naïve, vaccinated and infected animals

Sample source	Total no. samples	3B-cCLIA			3A + 3B-cCLIA			3ABC-bELISA			PrioCHECK® NSP		
		P^a^	N^b^	Accuracy rate	P^a^	N^b^	Accuracy rate	P^a^	N^b^	Accuracy rate	P^a^	N^b^	Accuracy rate
Naïve swine	310	1	309	99.68%	1	309	99.68%	2	308	99.35%	0	310	100.00%
Vaccinated swine with multiple-epitope recombinant vaccine	100	1	99	99.00%	1	99	99.00%	1	99	99.00%	0	100	100.00%
Infected swine	107	104	3	97.20%	105	2	98.13%	102	5	95.33%	91	16	85.05%
Naïve bovine	175	1	174	99.43%	1	174	99.43%	2	173	98.86%	0	175	100.00%
Infected bovine	70	67	3	95.71%	67	3	95.71%	66	4	94.29%	63	7	90.00%
Naïve sheep	61	1	60	98.36%	1	60	98.36%	2	59	96.72%	0	61	100.00%
Infected sheep	52	50	2	96.15%	50	2	96.15%	51	1	98.08%	48	4	92.31%
Vaccinated sows with commercial O/A divalent vaccine (3–15 times)	120	1	119	99.17%	1	119	99.17%	3	117	97.50%	0	120	100.00%
Vaccinated dairy cows with commercial O/A divalent vaccine (2–10 times)	129	12	117	90.70%	13	116	89.92%	19	110	85.27%	15	114	88.37%
Vaccinated sheep with O, A or Asia 1 univalent inactivated vaccine (1–3 times)	77	5	72	93.51%	4	73	94.81%	6	71	92.21%	2	75	97.40%
Swine in the filed	173	13	160		14 (13)	159 (159)	99.42%^c^	15 (11)	158 (156)	96.53% ^c^	9 (7)	164 (158)	95.37% ^c^

^a^Positive

^b^Negative

^c^The coincidence rate of PI value of 3B-cCLIA with those of 3A + 3B-cCLIA, 3ABC-bELISA, and PrioCHECK FMDV NSP ELISA in testing field sera collected from swine was 99.42% (172/173), 96.53% (167/173), and 95.37% (165/173), respectively

### Accuracy rates and diagnostic performances

These serum samples (n = 875) were also examined using two commercial diagnostic kits to evaluate and compare the accuracy of the test methods (Table 1). The accuracy rate of the 3B-cCLIA (97.20%, 95.71%, and 96.15%) was nearly equivalent to that of the 3ABC-bELISA (95.33%, 94.29%, and 98.08%) and better than that of the PrioCHECK FMDV NSP ELISA (85.05%, 90.00%, and 92.31%) for serum samples from infected animals. For the naïve serum samples, the accuracy rate of the 3B-cCLIA (99.51%, 99.43%, and 98.36%) was slightly higher than that of the 3ABC-bELISA (99.27%, 98.86%, and 96.72%) and lower than that of the PrioCHECK FMDV NSP ELISA (100%).

The accuracy rates of the 3B-cCLIA and PrioCHECK FMDV NSP ELISA using serum samples from sows inoculated 3–15 times were nearly equal to those of the same assays using serum samples from negative swine, but the accuracy rate of the 3ABC-bELISA was slightly lower (Table 1). However, the accuracy rates of the three assays using sera from dairy cows inoculated 2–10 times were significantly lower than those using sera from naïve cattle (namely, an increase in the false-positive rate). Similarly, the accuracy rates using sera from sheep vaccinated 1–3 times with a laboratory-made FMDV A, O, or Asia 1 univalent inactivated vaccine were lower than those using naïve sheep sera (Table 1). Furthermore, the rate of false positives increased with the increasing number of vaccinations in cattle and sheep (Table 2). To address this problem, a competitive CLIA (3A + 3B-cCLIA) using anti-3ABC polyclonal antibodies as the capture antibodies to capture purified 3ABC protein and two mAbs (2G5-HRP and 9E2-HRP) as the detection antibodies was developed to simultaneously detect antibodies against 3A and 3B (Additional file 1: Fig. S1). However, the accuracy rate of the 3A + 3B-cCLIA using sera from cattle and sheep vaccinated multiple times was lower than that using naïve sera (Table 1).

**Table 2:** Antibody to FMDV NSP in sera from cattle and sheep collected following repeated vaccination

Sample source	Total no. samples	3B-cCLIA			3A + 3B-cCLIA			3ABC-bELISA			PrioCHECK® NSP		
		P^a^	N^b^	False-positive rate	P^a^	N^b^	False-positive rate	P^a^	N^b^	False-positive rate	P^a^	N^b^	False-positive rate (%)
Vaccinated dairy cows with commercial O/A divalent vaccine (2 times)	30	1	29	3.33%	1	29	3.33%	1	29	3.33%	1	29	3.33
Vaccinated dairy cows with commercial O/A divalent vaccine (5 times)	33	2	31	6.06*	2	31	6.06*	3	30	9.09%	3	30	9.09%
Vaccinated dairy cows with commercial O/A divalent vaccine (10 times)	66	9	57	13.64%	10	56	15.15%	15	51	22.73%	11	55	16.67%
Vaccinated sheep with O, A or Asia 1 univalent inactivated vaccine (1 time)	29	0	29	0%	1	28	3.45%	0	29	0%	0	29	0%
Vaccinated sheep with O, A or Asia 1 univalent inactivated vaccine (2 times)	30	2	28	6.67%	1	29	3.33%	1	29	3.33%	0	30	0%
Vaccinated sheep with O, A or Asia 1 univalent inactivated vaccine (3 times)	18	3	15	16.67%	2	16	11.11%	5	13	27.78%	2	16	11.11%

^a^Positive

^b^Negative

### Comparison of early diagnostic performance

**Fig. 2:**
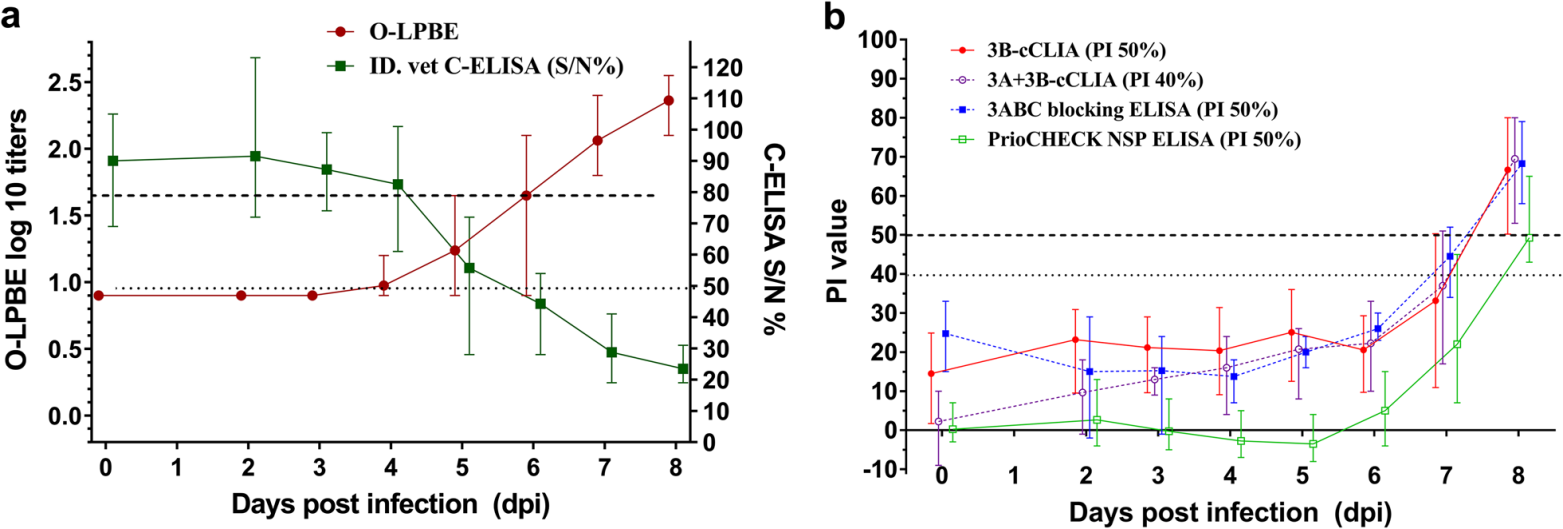
Early production of antibodies against FMDV SPs and NSPs detected in sera from experimentally challenged swine. **a** Thirty-two serum samples from four unvaccinated control swine after challenge with FMDV O/Mya98 were collected at 0 dpi and 2–8 dpi and tested using O-LPBE and ID Screen® FMD Type O Competition to detect anti-SP antibodies. The dashed lines _– – – – –_ and **……** represent the cut-off values of O-LPBE (reciprocal log 10 ≥ 1.65 was considered positive) and ID Screen® FMD Type O Competition (S/N ≤ 50% was considered positive), respectively. **b** The above thirty-two serum samples were also tested using the 3B-cCLIA, the 3A + 3B-cCLIA, and two commercial diagnostic kits (3ABC-bELISA and PrioCHECK FMDV NSP ELISA) to detect anti-NSP antibodies. The dashed line (_– – – – –_) represents the cut-off value of the 3B-cCLIA, 3ABC-bELISA, and PrioCHECK FMDV NSP ELISA (PI ≥ 50 was considered positive), and the dashed line (**……**) represents the cut-off value of the 3A + 3B-cCLIA (PI ≥ 40 was considered positive). The bar represents the range of values obtained by detecting sera from four swine at the given time

Seroconversion to NSPs and SPs was studied in four unvaccinated control swine post challenge using O-LPBE and ID Screen® FMD Type O Competition to detect anti-SP antibodies and using the 3B-cCLIA, 3A + 3B-cCLIA, 3ABC-bELISA, and PrioCHECK FMDV NSP ELISA to detect anti-NSP antibodies. Antibodies against SPs were first detected at 5 dpi, and all the sera examined were positive at 7 dpi (Fig. 2a). Antibodies against NSPs were detected at 7 dpi and identified as positive in all swine at 8 dpi, except in the PrioCHECK FMDV NSP ELISA (Fig. 2b). Therefore, seroconversion to NSPs occurred approximately 1–2 days later than that to SPs.

### Repeatability and stability of the 3B-cCLIA

**Fig. 3:**
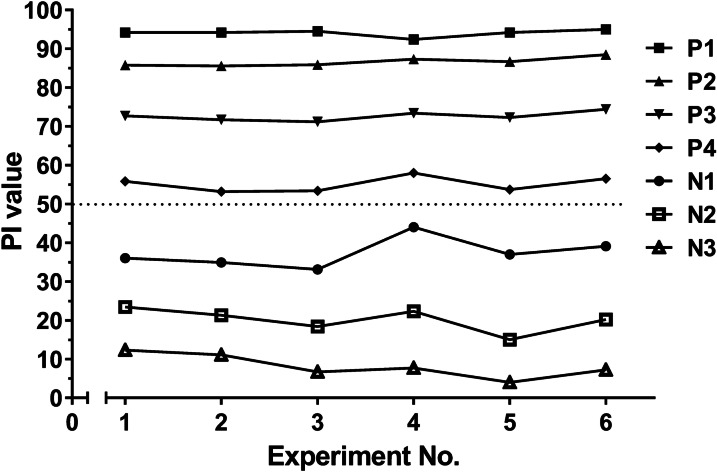
Intra- and interbatch repeatability performances of the 3B-cCLIA were assessed using four positive and three negative serum samples tested on different days on the same and different batch plates. The dashed line represents the cut-off value of the 3B-cCLIA

The assay was highly repeatable, as determined by four positive and three negative serum samples tested on different days on the same and different batch plates (Fig. 3).

Standard negative serum (P734) and standard positive serum (P51) were examined using 3B-cCLIA plates stored at 37 °C for 15 days or at 4 °C for 1 year. The CL values of P734 were > 6,000,000, and the PI value of P51 was > 80%. The findings showed that 3B-cCLIA plates may be preserved for up to 15 days at 37 °C and up to 1 year at 4 °C.

## Discussion

DIVA tests are important for serological surveillance, as they indicate whether vaccinated herds are infected with or free from FMD [1, 3, 17]. To avoid omitting animals infected with different serotypes, identifying conserved mAbs is important in the development of a diagnostic method for DIVA. The epitope “^23^EGPYAGPLE^31^” identified by the mAb 9E2 is located in 3B and is well conserved among the different serotypes of FMDV, which has been verified in a previous study [3]. Therefore, in this study, a competitive CLIA (3B-cCLIA) using the mAb 2G5 as the capture antibody to capture purified 3ABC protein and 9E2-HRP as the detection antibody was developed. The cut-off PI value of the 3B-cCLIA was determined to be 50% by testing panels of sera from different origins. Based on the cut-off PI of 50%, the Dsn (97.20%, 95.71%, and 96.15%) and Dsp (99.51%, 99.43%, and 98.36) were determined in naïve and infected swine, cattle and sheep, respectively (Fig. 1). The PrioCHECK FMDV NSP ELISA had good Dsp (accuracy rate 100%) for naïve sera but had low Dsn for infected sera when compared with the 3B-cCLIA and 3ABC-bELISA.

Although most NSPs are removed from the inactivated vaccine, residual NSPs remain [3] and can induce the production of the corresponding antibodies after repeated vaccination, especially in cattle [11, 12, 21–23]; this will interfere with the DIVA test and affect serological surveillance and evaluation of infection status [19]. This phenomenon was also confirmed in this study, in which dairy cows vaccinated multiple times with the traditional inactivated vaccine had a high false-positive rate on the 3B-cCLIA and two commercial diagnostic kits (Tables 1 and 2). In addition, the false-positive rate in sheep vaccinated three times also increased (Tables 1 and 2). However, the false-positive rate in sows vaccinated multiple times was almost equal to that of negative sera (Tables 1 and 2), similar to previous reports [12, 18]; this result may be due to different immune mechanisms in different species. The false-positive rate increased with the number of vaccinations. To address this problem, we attempted to use two mAbs recognizing NSP 3A and 3B to develop the 3A + 3B-cCLIA. In theory, only when the two antibodies are simultaneously present in serum is the serum considered positive; therefore, the assay should decrease the false-positive rate in vaccinated animals. However, the results showed that the 3A + 3B-cCLIA did not improve the false-positive rate (Tables 1 and 2).

Sera from some animals vaccinated multiple times are usually weakly positive. Therefore, increasing the cut-off value (compromising true sensitivity) is an approach to decrease the false-positive rate in vaccinated animals. Another approach is to establish a correlation between residual NSPs in inactivated vaccines with different rounds of purification and anti-NSP antibodies in different species that were inoculated different numbers of times with the corresponding inactivated vaccine, thereby confirming a threshold value for residual NSPs in inactivated vaccines to guide vaccine manufacturers in improving vaccine purity. In addition, the development and use of new FMD vaccines that do not contain NSP components, such as marker vaccines [24, 25], virus-like particle (VLP) vaccines [26], multiple-epitope recombinant vaccines [27], and synthetic peptide vaccines, could eliminate this challenge.

In this study, seroconversion to NSPs occurred later than that to SPs, similar to previous reports [11, 21]. The seroconversion time of the PrioCHECK FMDV NSP ELISA was later than those of the other three methods, which explains the lower Dsn of the PrioCHECK FMDV NSP ELISA in detecting sera from infected animals.

## Conclusion

A competitive CLIA that can rapidly detect antibodies against FMDV NSP 3B was developed in this study. The 3ABC-bELISA and PrioCHECK FMDV NSP ELISA use the blocking ELISA format and require washing twice in the process, while the 3B-cCLIA uses the competitive format, needs to be washed only once and requires only 15 min to obtain results. On the basis of the results of the study, the 3B-cCLIA had good Dsn, Dsp, repeatability and stability. Therefore, the 3B-cCLIA is a promising tool for rapidly diagnosing animals infected with FMDV in large-scale serological surveys. In addition, anti-NSP antibody detection methods are largely influenced by the purity of FMD vaccines and number of immunizations when assaying cattle and sheep, which interfere with DIVA.

## Supplementary Information

**Additional file 1: Figure S1** Receiver operating characteristic (ROC) analysis for the determination of the cut-off value of the 3A+3B-cCLIA. (a, c, e) Interactive dot diagram of the 3A+3B-cCLIA in testing sera from swine, cattle, and sheep. 0, negative serum samples (n = 410, 175, and 61); 1, positive serum samples (n = 107, 70, and 52). (b, d, and f) Each point on the ROC curve represents a sensitivity-specificity pair in testing the sera from swine, cattle, and sheep.

**Additional file 2: Table S1** Detailed information of serum samples used in the study.

## Data Availability

All data associated with this study are included in the paper and its Additional files 1 and 2.

## Abbreviations

FMD: Foot-and-mouth disease
FMDV: Foot-and-mouth disease virus
DIVA: Differentiation between infected and vaccinated animals
CLIA: Chemiluminescence immunoassay
NSP: Non-structural protein
SP: Structural protein
ELISA: Enzyme-linked immunosorbent assay
mAbs: Monoclonal antibodies
O-LPBE: Liquid-phase blocking ELISA of FMDV O
A-LPBE: Liquid-phase blocking ELISA of FMDV A
Dsp: Diagnostic specificity
Dsn: Diagnostic sensitivity
dpv: Days post vaccination
dpi: Days post infection
CSFV: Classical swine fever virus
SVA: Senecavirus A
PPV: Porcine parvovirus
PRRSV: Porcine reproductive and respiratory syndrome virus
PCV2: Porcine circovirus type 2
PI: Percentage inhibition
HRP: Horseradish peroxidase
N/P: The ratio of the CL values of standard negative serum to those of standard positive serum
ROC: Receiver operating characteristic
