# Supplementary figures and images for: Development of a competitive chemiluminescence immunoassay using a monoclonal antibody recognizing 3B of foot-and-mouth disease virus for the rapid detection of antibodies induced by FMDV infection
Source: Virol J. 2021 Sep 26;18:193. doi: 10.1186/s12985-021-01663-4 (PMC8474858; doi:10.1186/s12985-021-01663-4)

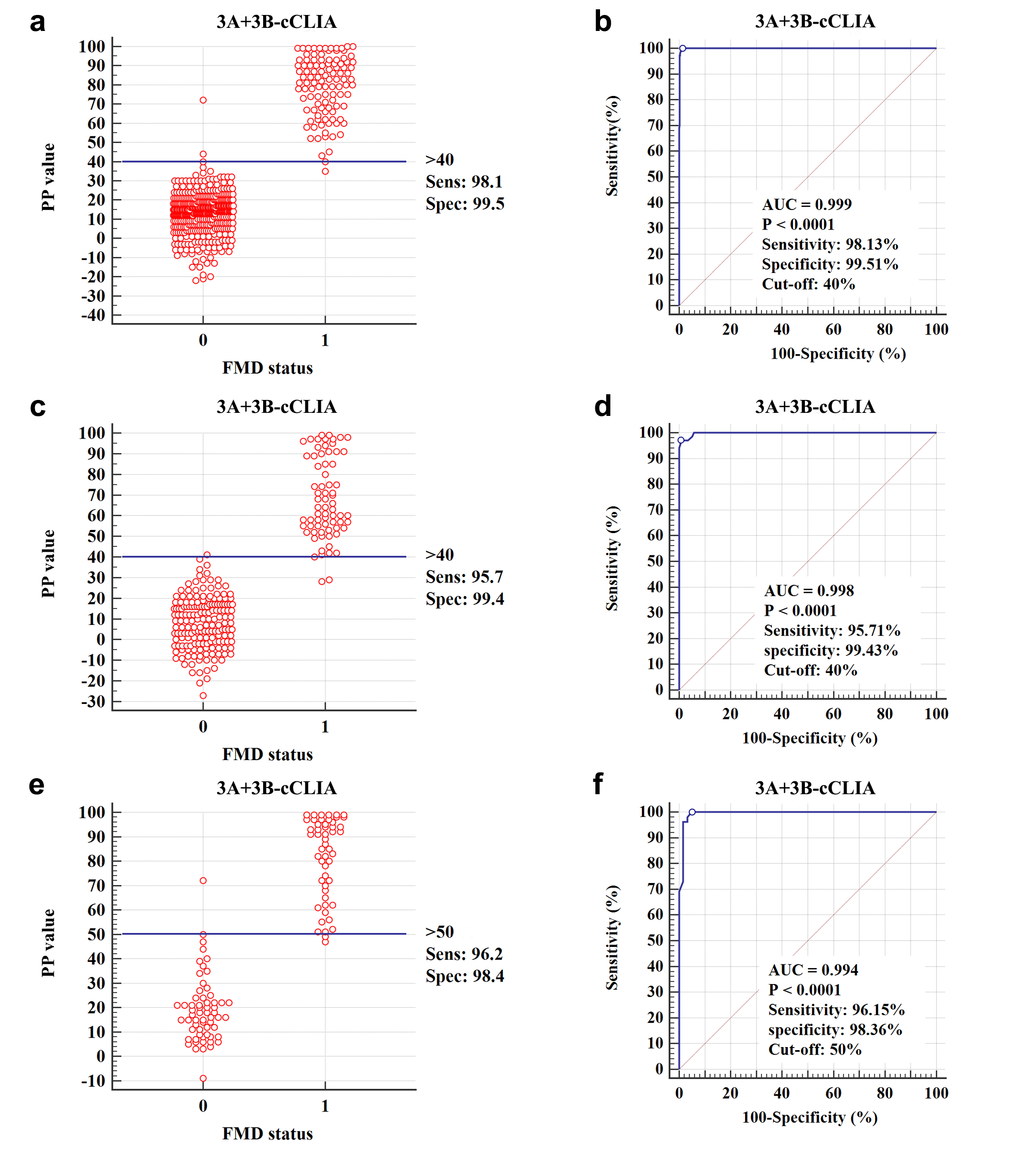

Supplement: Supplementary file 1 — Additional file 1: Figure S1 Receiver operating characteristic (ROC) analysis for the determination of the cut-off value of the 3A+3B-cCLIA. (a, c, e) Interactive dot diagram of the 3A+3B-cCLIA in testing sera from swine, cattle, and sheep. 0, negative serum samples (n = 410, 175, and 61); 1, positive serum samples (n = 107, 70, and 52). (b, d, and f) Each point on the ROC curve represents a sensitivity-specificity pair in testing the sera from swine, cattle, and sheep. [file 12985_2021_1663_MOESM1_ESM.tif]
