# Supplementary material for: Development of a competitive chemiluminescence immunoassay using a monoclonal antibody recognizing 3B of foot-and-mouth disease virus for the rapid detection of antibodies induced by FMDV infection
Source: Virol J. 2021 Sep 26;18:193. doi: 10.1186/s12985-021-01663-4 (PMC8474858; doi:10.1186/s12985-021-01663-4)
Supplement: Supplementary file 2 — Additional file 2: Table S1 Detailed information of serum samples used in the study. [file 12985_2021_1663_MOESM2_ESM.docx]

**Development of a competitive** **chemiluminescence immunoassay using a monoclonal antibody recognizing 3B of foot-and-mouth disease virus for the rapid detection of antibodies induced by** **FMDV infection**

Wei Liu, Guanglei Zhang, Sicheng Yang, Junhui Li, Zhan Gao, Sudan Ge, Huihui Yang, Junjun Shao^#^, Huiyun Chang^#^

State Key Laboratory of Veterinary Etiological Biology, National Foot-and-Mouth Diseases Reference Laboratory, Lanzhou Veterinary Research Institute, Chinese Academy of Agricultural Sciences, Lanzhou, Gansu Province, China

# Corresponding author: Huiyun Chang, [changhuiyun@caas.cn](mailto:changhuiyun@caas.cn)

Junjun Shao shaojunjun@caas.cn

### Development of 3A+3B-cCLIA using mAbs 2G5 and 9E2

The conditions of 2G5-cCLIA were optimized. Briefly, polyclonal antibodies against 3ABC were coated onto 96-well white plates at the concentrations of 0.5, 0.25, 0.125, and 0.0625 μg/mL and incubated overnight at 4 °C. After washing, purified 3ABC protein was diluted to 0.25 μg/mL and added to each well, and the plate was incubated for 1 h at 37 °C. After three washes with PBST, 200 μL of blocking buffer was added to each well and incubated for 2 h at 37 °C. Then, standard positive serum (P51) and standard negative serum (P734) were serially diluted at 1:2.5–1:10 dilutions, 50 μL of the serum was transferred to each well. Simultaneously, 50 μL of HRP-conjugated 2G5 (2G5-HRP) was added to each well at concentrations of 1, 0.5, 0.25, 0.125, 0.0625, and 0.03125 μg/mL, followed by incubation of the plate at room temperature. After washing five times, 100 μL of chemiluminescence (CL) substrate were added. After 5 min, the CL signals were measured. The optimum polyclonal antibody concentration, serum dilution, 2G5-HRP concentration, and incubation time were determined on the basis of the ratios of CL values of standard negative serum to those of standard positive serum (N/P).

Similarly, the conditions of 9E2-cCLIA were optimized with 9E2-HRP concentrations of 0.01, 0.005, 0.0025, 0.00125, and 0.000625 μg/mL.

In the 2G5-cCLIA and 9E2-cCLIA, the coating concentration of polyclonal antibodies was fixed at 0.25 μg/mL, the serum dilution was 1:2.5, the optimum concentration of 2G5-HRP was 0.0625–0.25 μg/mL, the optimum concentration of 9E2-HRP was 0.0025–0.01 μg/mL, and the reaction time was 10 min. The CL value of the 2G5-cCLIA was approximately equal to that of the 9E2-cCLIA, when concentrations of mAbs 2G5 and 9E2 were 0.17 μg/mL and 0.006 μg/mL, respectively. Therefore, 0.34 μg/mL 2G5-HRP were mix with 0.012 μg/mL 9E2-HRP in equal volumes referred to as mixed competitive mAbs in the 3A+3B-cCLIA.

### Cutoff value, Dsn, and Dsp

The cut-off values of the 3A+3B-cCLIA were determined by testing 875 serum samples from different origins. The Dsn and Dsp of swine, cattle, and sheep were estimate using MedCalc software.

The cut-off value of the 3A+3B-cCLIA in testing sera from swine and cattle was determined as 40%, and the Dsn was 98.13% and 95.71%, and Dsp was 99.51% and 99.43% in swine and cattle, respectively. The cut-off was determined as 50% when testing sera from sheep, and the Dsn and Dsp was 96.15% and 98.36%, respectively (Figure S1).


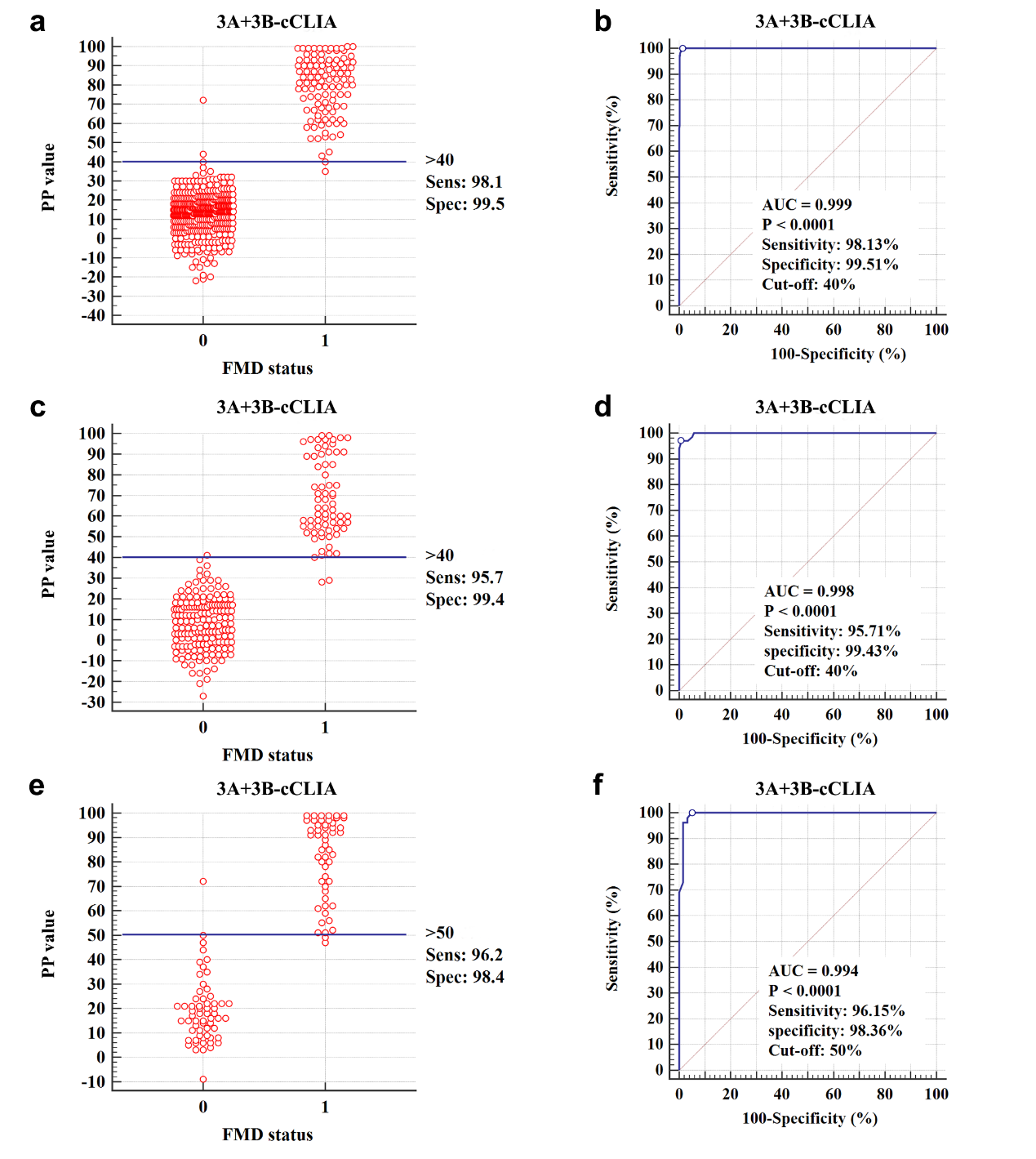
**Figure S1** Receiver operating characteristic (ROC) analysis for the determination of the cut-off value of the 3A+3B-cCLIA. (a, c, e) Interactive dot diagram of the 3A+3B-cCLIA in testing sera from swine, cattle, and sheep. 0, negative serum samples (n = 410, 175, and 61); 1, positive serum samples (n = 107, 70, and 52). (b, d, and f) Each point on the ROC curve represents a sensitivity-specificity pair in testing the sera from swine, cattle, and sheep

Table S1 Detailed information of serum samples used in the study

| Sample source | Total no. of samples | Time of samples collected | Purpose |
| --- | --- | --- | --- |
| Naïve swine | 310 |  | to estimate the cut-off value and Dsp in swine |
| Vaccinated swine with FMDV O univalent multiple-epitope recombinant vaccine | 100 | 21 dpv |  |
| Infected swine with O/Mya98 or A/GDMM/2013 | 107 | 7–25 dpi | to estimate the cut-off value and Dsn in swine |
| Unvaccinated control swine experimentally challenged with FMDV O/Mya98 | 32 | 0 dpi and 2–8 dpi | to estimate the early diagnostic performance and seroconversion |
| Vaccinated sows 3–15 times with commercial O/A divalent inactivated vaccine | 120 | 7–10 days post vaccination | to evaluate the diagnostic performances and verify the false-positive phenomenon in swine |
| Swine suspected of FMDV infection in the field | 173 |  | to compare the coincidence rates |
| Infected swine with SVA, CSFV, PRRSV, PPV and PCV2 | 6 |  | to evaluate the cross-reaction with sera from other virus-infected swine |
| Naïve cattle | 175 |  | to estimate the cut-off value and Dsp in cattle |
| Infected cattle with O/Mya98 or A/GDMM/2013 | 70 | 8–20 dpi | to estimate the cut-off value and Dsn in cattle |
| Vaccinated dairy cows 2, 5, or 10 times with commercial O/A divalent inactivated vaccine | 129 | 7–10 days post vaccination | to evaluate the diagnostic performances and verify the false-positive phenomenon in cattle |
| Naïve sheep | 61 |  | to estimate the cut-off value and Dsp in sheep |
| Infected sheep in the field | 52 |  | to estimate the cut-off value and Dsn in sheep |
| Vaccinated sheep 1–3 times with lab-made O/Mya98/BY/2010, A/AF72, or Asia 1/JS05 univalent inactivated vaccine | 77 | 7 days post vaccination | to evaluate the diagnostic performances and verify the false-positive phenomenon in sheep |

Dsp, diagnostic specificity; Dsn, diagnostic sensitivity; dpv, days post vaccination; dpi, days post infection
